# The geriatric nutritional risk index as a prognostic risk factor for critically ill patients with atrial fibrillation: a retrospective study based on MIMIC-IV and local hospital cohort external validation

**DOI:** 10.3389/fnut.2026.1837424

**Published:** 2026-07-06

**Authors:** HongLi Luo, YuXuan Yan, LiJun Guo, WeiXian Xie, Heng Li, Weichao Li

**Affiliations:** 1 Department of Anesthesiology, Affiliated Qingyuan Hospital (Qingyuan People’s Hospital), Guangzhou Medical University, Qingyuan, Guangdong, China; 2 Department of Anesthesiology and Surgical Services, The First People Hospital Of Lanzhou, Lanzhou, Gansu, China

**Keywords:** atrial fibrillation, critically ill patients, external validation, geriatric nutritional risk index, mortality

## Abstract

**Background:**

Malnutrition is known to worsen outcomes in many diseases. This study explored its effect on prognosis in critically ill patients with atrial fibrillation (AF).

**Methods:**

A total of 4,564 patients were studied. Their nutritional status was assessed using the Geriatric Nutritional Risk Index (GNRI), and they were divided into four groups based on GNRI quartiles, with a median of 100.23 and interquartile range of 90.31–111.27. The primary outcome was 28-day mortality. Kaplan–Meier analysis evaluated survival differences among the GNRI groups, and a multivariate Cox model assessed GNRI’s independent effect on mortality risk. A restricted cubic spline (RCS) analysis explored potential non-linear associations between GNRI and 28-day mortality.

**Results:**

There is a negative correlation between GNRI and 28-day mortality, a finding supported by a log-rank test result with *p* < 0.001. According to the multivariate analysis, higher GNRI values were independently associated with a lower risk of 28-day mortality (quartile 4 compared to quartile 1, the hazard ratio (HR) was 0.78 with a 95% confidence interval (CI) of 0.62–0.97, and *p* = 0.029). Subgroup analysis showed no association in those with obesity, or in cardiovascular ICUs or post-cardiac surgery settings. No link was found in patients with a history of AF or on CRRT. However, the association was consistently seen in septic patients with new-onset AF. Additionally, the RCS analysis revealed a non-linear relationship between GNRI and 28-day mortality, showing that as GNRI increased, the risk of 28-day mortality decreased among patients. Findings from external validation cohorts also confirm this relationship.

**Conclusion:**

Among critically ill patients, elevated GNRI scores are associated with improved outcomes in patients with new-onset AF, but this association is not present in patients with preexisting AF. In the subgroup of septic patients with new-onset AF, GNRI may not reliably predict clinical prognosis, emphasizing the need for further validation prior to clinical use. The GNRI provides a straightforward, unbiased assessment of both nutritional status and systemic inflammation burden.

## Highlights

- *Question*: Does elevated Geriatric Nutritional Risk Index (GNRI) correlate with decreased mortality risk among critically ill patients with atrial fibrillation (AF)?
- *Study findings*: Using two large ICU databases for a multicenter cohort study, we found that elevated GNRI levels were associated with a significantly lower adjusted risk of mortality relative to patients with lower GNRI values. However, this correlation lacked statistical significance in subgroups including obese patients, those admitted to cardiovascular ICUs, and post-cardiac surgery patients. Additionally, no such association was observed in patients with a history of AF or undergoing CRRT. In the subgroup of patients with sepsis and new-onset atrial fibrillation, the association between GNRI and mortality was not replicated in the external validation cohort.
- *Implications*: Overall, these results imply that elevated GNRI might be linked to decreased mortality among critically ill patients presenting with new-onset AF, rather than those with a prior history of AF. While these observational findings support a potential link between nutritional status and outcomes, randomized controlled trials are necessary to formally establish a causal relationship before clinical translation can be considered.

## Introduction

Atrial fibrillation (AF) is observed in approximately 15.6% of ICU admissions (1, 2), with incidence rising to 10–46% among patients with septic shock (3, 4). For critically ill patients, AF precipitates cardiovascular instability and correlates with elevated thromboembolic risk, higher mortality rates, extended ICU hospitalization duration, and increased healthcare expenditures (5, 6). New-onset atrial fibrillation (NOAF) in intensive care units refers to AF occurring in individuals without a prior history of documented AF (7, 8).

Hospital-based AF management guidelines demonstrate limited relevance to the care of critically ill patients with AF (9). To date, no consensus-driven protocols for ICU-AF exist beyond standard interventions: beta-blockers for heart rate control, amiodarone for rhythm management, and electrical cardioversion. Inconsistent strategies for ICU management of AF involve magnesium, potassium, the selective L-type calcium channel blocker diltiazem, hydrocortisone, digoxin, propafenone, and flecainide. This evidence gap stems largely from the unique mechanistic profile of ICU-AF, distinct from hospital-acquired AF, including inflammation, sympathetic nervous system overactivation (attributable to disease pathophysiology and vasoactive drug administration), electrolyte imbalances (hypokalemia, hypomagnesemia, hypocalcemia), asynchronous mechanical ventilation, reluctance to anticoagulate in ICU settings, greater illness severity, volume-overload or hypovolemia, and pre-critical illness comorbidities (10).

Nutritional impairment encompasses acute, subacute, or chronic deviations from optimal nutritional status. Defined by variable degrees of over- or undernutrition, with or without associated inflammation, this state disrupts body composition and impairs physical function (11). Nutritional impairment is increasingly recognized as a contributor to cardiovascular disease progression (12, 13), including inflammatory processes, atherosclerosis, and arterial calcification (14). Underlying pathways likely involve proinflammatory cytokine upregulation, increased endotoxin translocation, and reduced detoxification capacity (15). Clinically, up to 40% of cardiovascular disease (CVD) cases may be linked to suboptimal nutritional status (16). Notably, CVD patients commonly experience malnutrition due to impaired metabolic efficiency, heightened catabolic demands, and gastrointestinal dysfunction (17), with targeted nutritional support shown to improve patient outcomes and quality of life (18, 19).

The Geriatric Nutritional Risk Index (GNRI) is a validated, user-friendly tool for nutritional status assessment. Numerous studies have demonstrated that GNRI-measured nutritional status is closely associated with the onset and clinical course of critically ill patients with related diseases, including sepsis (20, 21), acute respiratory distress syndrome (ARDS) (22), and acute kidney injury (AKI) (23, 24). Despite divergent pathophysiology and clinical management strategies for community-acquired versus ICU-acquired AF, even though GNRI is recognized as a validated risk factor for post-acute myocardial infarction new-onset AF (25), the prognostic implications of nutritional status in ICU patients with AF remain incompletely understood. Therefore, this study was designed to examine the prognostic value of GNRI in critically ill patients diagnosed with AF.

## Methods

### Data acquisition and study design

For clarity and consistency, data were retrieved from the public Medical Information Mart for Intensive Care IV (MIMIC-IV) database. MIMIC-IV access was facilitated through PostgreSQL, with variables extracted using SQL queries from the MIMIC GitHub repository. MIMIC-IV v3.0 covers 53,150 individuals and 94,458 ICU admissions at Beth Israel Deaconess Medical Center during 2008–2022 (26). Owing to the dataset’s anonymization, ethical boards at Beth Israel Deaconess Medical Center (2001-P-001699/14) and the Massachusetts Institute of Technology (No. 0403000206) approved the waiver of informed consent. We performed an additional external validation cohort by utilizing the Clinical Data Warehouse integrated with the Electronic Medical Record System at Qingyuan People’s Hospital (QYPH). This data repository is a continuously maintained and periodically refreshed database that holds de-identified information gathered from standard clinical practice at QYPH, a prominent tertiary academic healthcare facility located in Qingyuan, Guangdong Province, China. No connections were made with any outside data resources. The research encompassed all adult patients 18 years of age or older who were diagnosed with AF and underwent sequential treatment from January 2019 through July 2024. The ethics committee at QYPH granted approval for the study and exempted the need for obtaining written informed consent. Observational research reporting in this study followed STROBE guidelines.

### Definitions and outcome measures

The GNRI calculation equation is as follows: GNRI = (14.89 × serum albumin [g/dL]) + (41.7 × actual BMI/ideal BMI) (27). Ideal BMI was specified as 22 kg/m^2^, and the actual-to-ideal BMI ratio was set to 1 for patients with BMI above this threshold (28). All patients were categorized into four quartile groups by GNRI values: Quartile 1 (GNRI < 90.31), Quartile 2 (90.31 ≤ GNRI < 100.23), Quartile 3 (100.23 ≤ GNRI < 111.28), Quartile 4 (GNRI ≥ 111.28). NOAF was identified using validated nurse-documented cardiac rhythm data extracted from the MIMIC-IV database (29, 30). The primary outcome measure was 28-day mortality, while 28-day ICU mortality served as the secondary endpoint. Obesity was defined as a BMI of 30 kg/m^2^ or higher, consistent with standard World Health Organization criteria. Exclusion criteria were: age <18 years, missing BMI data, or presence of other medical data irregularities (e.g., SpO₂ > 100%). Furthermore, for patients with multiple ICU admissions, only the initial admission records were included in the analysis.

### Data collection and definitions

Utilizing PostgreSQL (v13.7.2) and Navicat Premium (v16.0), data extraction was executed through structured query language (SQL). The following variables were extracted in the study: age; BMI; gender; race and ethnicity; initial vital signs [HR (heart rate), RR (respiratory rate), SpO₂ level, and temperature]; ICU type (Cardiovascular ICU, Coronary Care Unit, Medical ICU, and Surgical ICU); Charlson Comorbidity Index; SOFA score; APS III score; comorbidities (Sepsis, Cardiovascular disease, Chronic liver disease, Chronic neurologic disease, Diabetes, Cancer, Chronic Kidney Disease (CKD), and Chronic lung disease); medications (Electrical Cardioversion, rate control medication, Dexmedetomidine, and rhythm control medication); life support interventions (mechanical ventilation and continuous renal replacement therapy [CRRT]); and baseline laboratory tests (white blood cell count, platelet count, hemoglobin level, PH, pCO_2_ level, pO_2_ level, lactate level, albumin, ALT, AST, urea nitrogen level, creatinine level, sodium level, potassium level, calcium level, chloride level, glucose level, magnesium level, and phosphorus level) (all detailed in Table 1). Rate control medication consisted of diltiazem, esmolol, metoprolol, and digoxin. Rhythm control medication consisted of amiodarone, magnesium, sotalol, dronedarone, propafenone, and flecainide.

**Table 1:** Patient baseline characteristics in the MIMIC-IV cohort stratified by GNRI quartiles.

Variable	[Median (IQR)]					
	Overall	Q1 GNRI < 90.31	Q2 90.31 ≤ GNRI < 100.23	Q3 100.23 ≤ GNRI < 111.28	Q4 GNRI ≥ 111.28	*p*
	*n* = 4,564	*n* = 1,140	*n* = 1,141	*n* = 1,142	*n* = 1,141	
Age, ys	74.00 [66.00, 82.00]	77.00 [69.00, 85.00]	75.00 [67.00, 83.00]	74.00 [67.00, 81.75]	71.00 [63.00, 78.00]	<0.001
Gender (%)						0.003
Female	1,653 (36.2)	447 (39.2)	385 (33.7)	381 (33.4)	440 (38.6)	
Male	2,911 (63.8)	693 (60.8)	756 (66.3)	761 (66.6)	701 (61.4)	
Race (%)						<0.001
Asian	94 (2.1)	44 (3.9)	32 (2.8)	13 (1.1)	5 (0.4)	
Black	201 (4.4)	51 (4.5)	51 (4.5)	42 (3.7)	57 (5.0)	
Hispanic	93 (2.0)	17 (1.5)	27 (2.4)	25 (2.2)	24 (2.1)	
Other/unknown	876 (19.2)	214 (18.8)	204 (17.9)	229 (20.1)	229 (20.1)	
White	3,300 (72.3)	814 (71.4)	827 (72.5)	833 (72.9)	826 (72.4)	
Years admitted (%)						0.049
2008–2019	3,504 (76.8)	902 (79.1)	847 (74.2)	882 (77.2)	873 (76.5)	
2020–2022	1,060 (23.2)	238 (20.9)	294 (25.8)	260 (22.8)	268 (23.5)	
Obesity (%)						<0.001
No	2,722 (59.6)	1,066 (93.5)	908 (79.6)	604 (52.9)	144 (12.6)	
Yes	1,842 (40.4)	74 (6.5)	233 (20.4)	538 (47.1)	997 (87.4)	
Heart rate, beats/min	81.46 [73.92, 92.13]	81.31 [73.41, 92.75]	81.30 [73.42, 92.06]	80.90 [74.08, 91.11]	82.10 [75.12, 92.52]	0.375
Mean artery pressure, mm Hg	76.19 [70.08, 80.25]	76.19 [69.74, 80.72]	76.19 [70.14, 80.33]	76.19 [70.15, 79.94]	76.19 [70.47, 80.00]	0.873
Respiratory rate, breaths/min	18.46 [16.40, 21.00]	18.38 [16.21, 21.00]	18.24 [16.28, 20.79]	18.48 [16.38, 20.98]	18.79 [16.81, 21.19]	0.002
SpO_2_ level, %	97.34 [95.94, 98.48]	97.59 [96.14, 98.63]	97.40 [96.09, 98.62]	97.38 [95.86, 98.44]	97.01 [95.71, 98.25]	<0.001
Temperature, °C	36.69 [36.59, 36.92]	36.69 [36.55, 36.91]	36.69 [36.57, 36.89]	36.69 [36.61, 36.90]	36.69 [36.63, 36.96]	<0.001
ICU types (%)						<0.001
Cardiovascular ICU	2029 (44.5)	417 (36.6)	527 (46.2)	546 (47.8)	539 (47.2)	
Coronary care unit	750 (16.4)	205 (18.0)	195 (17.1)	190 (16.6)	160 (14.0)	
Medical ICU	569 (12.5)	157 (13.8)	130 (11.4)	130 (11.4)	152 (13.3)	
Other	118 (2.6)	42 (3.7)	20 (1.8)	31 (2.7)	25 (2.2)	
Surgical ICU	1,098 (24.1)	319 (28.0)	269 (23.6)	245 (21.5)	265 (23.2)	
SOFA score	5.00 [3.00, 8.00]	5.00 [3.00, 7.00]	5.00 [3.00, 7.00]	5.00 [3.00, 8.00]	5.00 [3.00, 8.00]	0.008
Charlson index	5.00 [4.00, 7.00]	6.00 [4.00, 8.00]	5.00 [4.00, 7.00]	6.00 [4.00, 8.00]	5.00 [4.00, 7.00]	<0.001
APS III score	42.00 [31.00, 58.00]	43.50 [33.00, 58.00]	41.00 [31.00, 56.00]	42.00 [31.00, 58.00]	42.00 [31.00, 58.00]	0.087
Life support measures (%)						
CRRT						<0.001
No	4,273 (93.6)	1,093 (95.9)	1,075 (94.2)	1,064 (93.2)	1,041 (91.2)	
Yes	291 (6.4)	47 (4.1)	66 (5.8)	78 (6.8)	100 (8.8)	
Ventilation						<0.001
No	482 (10.6)	164 (14.4)	125 (11.0)	97 (8.5)	96 (8.4)	
Yes	4,082 (89.4)	976 (85.6)	1,016 (89.0)	1,045 (91.5)	1,045 (91.6)	
Vasopressor						<0.001
No	1,309 (28.7)	394 (34.6)	322 (28.2)	309 (27.1)	284 (24.9)	
Yes	3,255 (71.3)	746 (65.4)	819 (71.8)	833 (72.9)	857 (75.1)	
Cardiac surgery (%)						<0.001
No	3,006 (65.9)	840 (73.7)	736 (64.5)	712 (62.3)	718 (62.9)	
Yes	1,558 (34.1)	300 (26.3)	405 (35.5)	430 (37.7)	423 (37.1)	
Comorbidity (%)						
Sepsis						0.439
No	1852 (40.6)	453 (39.7)	485 (42.5)	464 (40.6)	450 (39.4)	
Yes	2,712 (59.4)	687 (60.3)	656 (57.5)	678 (59.4)	691 (60.6)	
Cardiovascular disease						0.714
No	655 (14.4)	164 (14.4)	159 (13.9)	175 (15.3)	157 (13.8)	
Yes	3,909 (85.6)	976 (85.6)	982 (86.1)	967 (84.7)	984 (86.2)	
Chronic liver disease						0.449
No	4,381 (96.0)	1,098 (96.3)	1,101 (96.5)	1,095 (95.9)	1,087 (95.3)	
Yes	183 (4.0)	42 (3.7)	40 (3.5)	47 (4.1)	54 (4.7)	
Chronic neurologic disease						0.372
No	4,061 (89.0)	999 (87.6)	1,017 (89.1)	1,026 (89.8)	1,019 (89.3)	
Yes	503 (11.0)	141 (12.4)	124 (10.9)	116 (10.2)	122 (10.7)	
Chronic lung disease						0.015
No	3,105 (68.0)	734 (64.4)	804 (70.5)	787 (68.9)	780 (68.4)	
Yes	1,459 (32.0)	406 (35.6)	337 (29.5)	355 (31.1)	361 (31.6)	
Diabetes						<0.001
No	3,021 (66.2)	861 (75.5)	797 (69.9)	746 (65.3)	617 (54.1)	
Yes	1,543 (33.8)	279 (24.5)	344 (30.1)	396 (34.7)	524 (45.9)	
Cancer						<0.001
No	3,720 (81.5)	909 (79.7)	908 (79.6)	916 (80.2)	987 (86.5)	
Yes	844 (18.5)	231 (20.3)	233 (20.4)	226 (19.8)	154 (13.5)	
CKD						0.03
No	3,443 (75.4)	871 (76.4)	891 (78.1)	842 (73.7)	839 (73.5)	
Yes	1,121 (24.6)	269 (23.6)	250 (21.9)	300 (26.3)	302 (26.5)	
New-onset AF						0.009
No	1,128 (24.7)	317 (27.8)	293 (25.7)	263 (23.0)	255 (22.3)	
Yes	3,436 (75.3)	823 (72.2)	848 (74.3)	879 (77.0)	886 (77.7)	
Medications (%)						
Diuretic						<0.001
No	982 (21.5)	320 (28.1)	249 (21.8)	226 (19.8)	187 (16.4)	
Yes	3,582 (78.5)	820 (71.9)	892 (78.2)	916 (80.2)	954 (83.6)	
Insulin						0.048
No	2,870 (62.9)	682 (59.8)	714 (62.6)	729 (63.8)	745 (65.3)	
Yes	1,694 (37.1)	458 (40.2)	427 (37.4)	413 (36.2)	396 (34.7)	
Acetaminophen						0.448
No	2,811 (61.6)	687 (60.3)	720 (63.1)	712 (62.3)	692 (60.6)	
Yes	1753 (38.4)	453 (39.7)	421 (36.9)	430 (37.7)	449 (39.4)	
AF interventions						
Rate control medication						0.083
No	726 (15.9)	203 (17.8)	188 (16.5)	175 (15.3)	160 (14.0)	
Yes	3,838 (84.1)	937 (82.2)	953 (83.5)	967 (84.7)	981 (86.0)	
Rhythm control medication						0.002
No	525 (11.5)	161 (14.1)	139 (12.2)	111 (9.7)	114 (10.0)	
Yes	4,039 (88.5)	979 (85.9)	1,002 (87.8)	1,031 (90.3)	1,027 (90.0)	
Cardioversion						0.351
No	4,380 (96.0)	1,102 (96.7)	1,092 (95.7)	1,088 (95.3)	1,098 (96.2)	
Yes	184 (4.0)	38 (3.3)	49 (4.3)	54 (4.7)	43 (3.8)	
Dexmedetomidine						<0.001
No	3,548 (77.7)	929 (81.5)	911 (79.8)	891 (78.0)	817 (71.6)	
Yes	1,016 (22.3)	211 (18.5)	230 (20.2)	251 (22.0)	324 (28.4)	
Statins						0.04
No	3,995 (87.5)	1,024 (89.8)	981 (86.0)	994 (87.0)	996 (87.3)	
Yes	569 (12.5)	116 (10.2)	160 (14.0)	148 (13.0)	145 (12.7)	
Phosphorus						0.543
No	3,039 (66.6)	745 (65.4)	759 (66.5)	757 (66.3)	778 (68.2)	
Yes	1,525 (33.4)	395 (34.6)	382 (33.5)	385 (33.7)	363 (31.8)	
Magnesium sulfate						0.004
No	537 (11.8)	164 (14.4)	141 (12.4)	115 (10.1)	117 (10.3)	
Yes	4,027 (88.2)	976 (85.6)	1,000 (87.6)	1,027 (89.9)	1,024 (89.7)	
Potassium						0.041
No	404 (8.9)	123 (10.8)	102 (8.9)	91 (8.0)	88 (7.7)	
Yes	4,160 (91.1)	1,017 (89.2)	1,039 (91.1)	1,051 (92.0)	1,053 (92.3)	
Calcium						<0.001
No	963 (21.1)	288 (25.3)	241 (21.1)	210 (18.4)	224 (19.6)	
Yes	3,601 (78.9)	852 (74.7)	900 (78.9)	932 (81.6)	917 (80.4)	
Iron						0.693
No	4,220 (92.5)	1,052 (92.3)	1,053 (92.3)	1,065 (93.3)	1,050 (92.0)	
Yes	344 (7.5)	88 (7.7)	88 (7.7)	77 (6.7)	91 (8.0)	
Laboratory tests						
GNRI	100.23 [90.31, 111.27]	83.79 [78.78, 87.36]	95.57 [92.93, 97.80]	105.27 [102.57, 108.13]	119.64 [114.88, 127.26]	<0.001
White blood cell count, /μL	11.93 [8.90, 15.40]	11.24 [8.40, 14.70]	11.53 [8.45, 14.77]	12.30 [9.43, 15.62]	12.67 [9.37, 16.50]	<0.001
Platelet count, ×10^3^/μL	165.67 [127.19, 216.00]	169.00 [123.29, 220.00]	161.33 [123.33, 208.50]	162.08 [126.50, 210.92]	174.33 [135.50, 220.50]	0.001
Hemoglobin level, g/dL	10.10 [8.80, 11.70]	10.10 [8.80, 11.60]	10.10 [8.90, 11.70]	10.10 [8.70, 11.70]	10.20 [8.80, 11.80]	0.551
PH	7.37 [7.35, 7.40]	7.37 [7.36, 7.41]	7.37 [7.35, 7.40]	7.37 [7.35, 7.40]	7.37 [7.34, 7.40]	<0.001
pCO_2_ level, mm Hg	41.54 [38.33, 43.19]	41.54 [37.00, 42.00]	41.54 [37.88, 42.50]	41.54 [38.44, 43.17]	41.54 [39.50, 45.00]	<0.001
pO_2_ level, mm Hg	176.23 [116.00, 230.00]	176.23 [112.25, 233.63]	176.23 [128.00, 233.30]	176.23 [118.21, 233.25]	176.23 [107.67, 221.33]	0.001
Lactate level, mg/dL	2.14 [1.53, 2.30]	2.20 [1.55, 2.27]	2.13 [1.55, 2.30]	2.13 [1.50, 2.31]	2.08 [1.50, 2.35]	0.871
Albumin, g/dL	3.10 [2.70, 3.50]	2.50 [2.20, 2.80]	3.00 [2.70, 3.40]	3.30 [3.00, 3.70]	3.60 [3.20, 4.00]	<0.001
ALT, U/L	31.00 [16.00, 84.00]	32.00 [16.00, 88.00]	30.00 [16.00, 86.00]	31.00 [16.00, 88.00]	30.00 [17.00, 78.00]	0.563
AST, U/L	43.00 [23.00, 124.00]	46.00 [25.00, 134.25]	41.00 [23.00, 115.00]	43.00 [24.00, 123.50]	41.00 [22.00, 123.00]	0.036
Sodium level, mEq/L	138.33 [136.00, 140.50]	138.50 [136.00, 141.00]	138.00 [136.00, 140.33]	138.33 [136.00, 140.40]	138.50 [136.00, 140.67]	0.18
Creatinine level, mg/dL	1.00 [0.80, 1.50]	1.00 [0.80, 1.50]	1.00 [0.80, 1.40]	1.00 [0.80, 1.50]	1.00 [0.80, 1.50]	0.011
Ureanitrogen level, mg/dL	20.00 [15.00, 32.00]	21.00 [15.00, 34.00]	20.00 [14.00, 30.00]	20.00 [15.00, 31.00]	21.00 [15.00, 33.00]	0.142
Potassium level, mEq/L	4.29 [3.95, 4.60]	4.22 [3.90, 4.55]	4.25 [3.93, 4.60]	4.30 [4.00, 4.63]	4.30 [4.00, 4.63]	<0.001
Calcium level, mg/dL	8.35 [8.00, 8.65]	8.35 [8.00, 8.65]	8.35 [8.00, 8.63]	8.35 [8.07, 8.67]	8.35 [8.00, 8.65]	0.621
Chloride level, mEq/L	105.33 [101.50, 108.33]	105.50 [101.47, 108.50]	105.50 [101.67, 108.50]	105.31 [101.67, 108.31]	105.00 [101.00, 108.00]	0.037
Glucose level, mg/dL	126.16 [108.00, 150.54]	124.00 [105.67, 146.08]	123.67 [106.00, 148.00]	126.59 [109.00, 149.88]	131.00 [111.00, 159.50]	<0.001
Magnesium level, mEq/L	2.10 [1.90, 2.50]	2.10 [1.80, 2.40]	2.10 [1.90, 2.40]	2.10 [1.90, 2.50]	2.19 [1.90, 2.50]	<0.001
Phosphorus level, mEq/L	3.77 [3.10, 4.10]	3.70 [3.10, 4.10]	3.75 [3.10, 4.00]	3.77 [3.15, 4.05]	3.77 [3.20, 4.20]	0.001

Continuous variables are expressed as mean ± SD or median (IQR). Categorical variables are expressed as count (proportion). P values were generated via one-way ANOVA, Kruskal-Wallis H test, or Chi-square test to assess variable differences between GNRI quartile groups. GNRI, Geriatric Nutrition Risk Index; APS III, Acute Physiology Score III; CKD, Chronic kidney disease; CRRT, continuous renal replacement therapy; SOFA, sequential organ failure assessment; CKD, Chronic kidney disease; AF, atrial fibrillation; ALT, alanine aminotransferase; AST, aspartate aminotransferase.

### Subgroup analyses

We conducted pre-defined interaction analyses to explore the relationship between GNRI levels and 28-day mortality, stratifying patients by the following clinical characteristics: age (<65 vs. ≥ 65 years), sex, race/ethnicity (Black vs. non-Black), presence or absence of obesity, SOFA rating (<6 vs. ≥ 6), mechanical ventilation use, CKD, and vasopressor administration.

### Statistical analysis

The sample size for the MIMIC-IV cohort was determined based on the available data in the MIMIC-IV database, without conducting a prior statistical power analysis. For the local hospital cohort, the sample size estimation was derived from a 28-day mortality prevalence of about 16.3%, as observed in the MIMIC-IV cohort. The study aimed to include around 1,000 patients with AF, anticipating approximately 170 cases of 28-day mortality. With a C-statistic target of 0.8 and 21 candidate predictor parameters, a minimum of 1,079 subjects was required to develop the model. This calculation assumed an acceptable difference of 0.05 in the apparent adjusted R^2^ and a margin of error of 0.05 in estimating the intercept. Continuous variables were reported as mean ± standard deviation (mean ± SD) or median and interquartile range (IQR), while categorical variables were expressed as counts and percentages. For intergroup comparisons, *p* values were generated via one-way ANOVA, Kruskal-Wallis H test, or Chi-square test to assess variable differences among GNRI quartile groups. Missing data were handled through the missForest algorithm in R: variables with <30% missingness had values imputed, while those with >30% missing data were excluded from the analysis (31).

**Figure 1:**
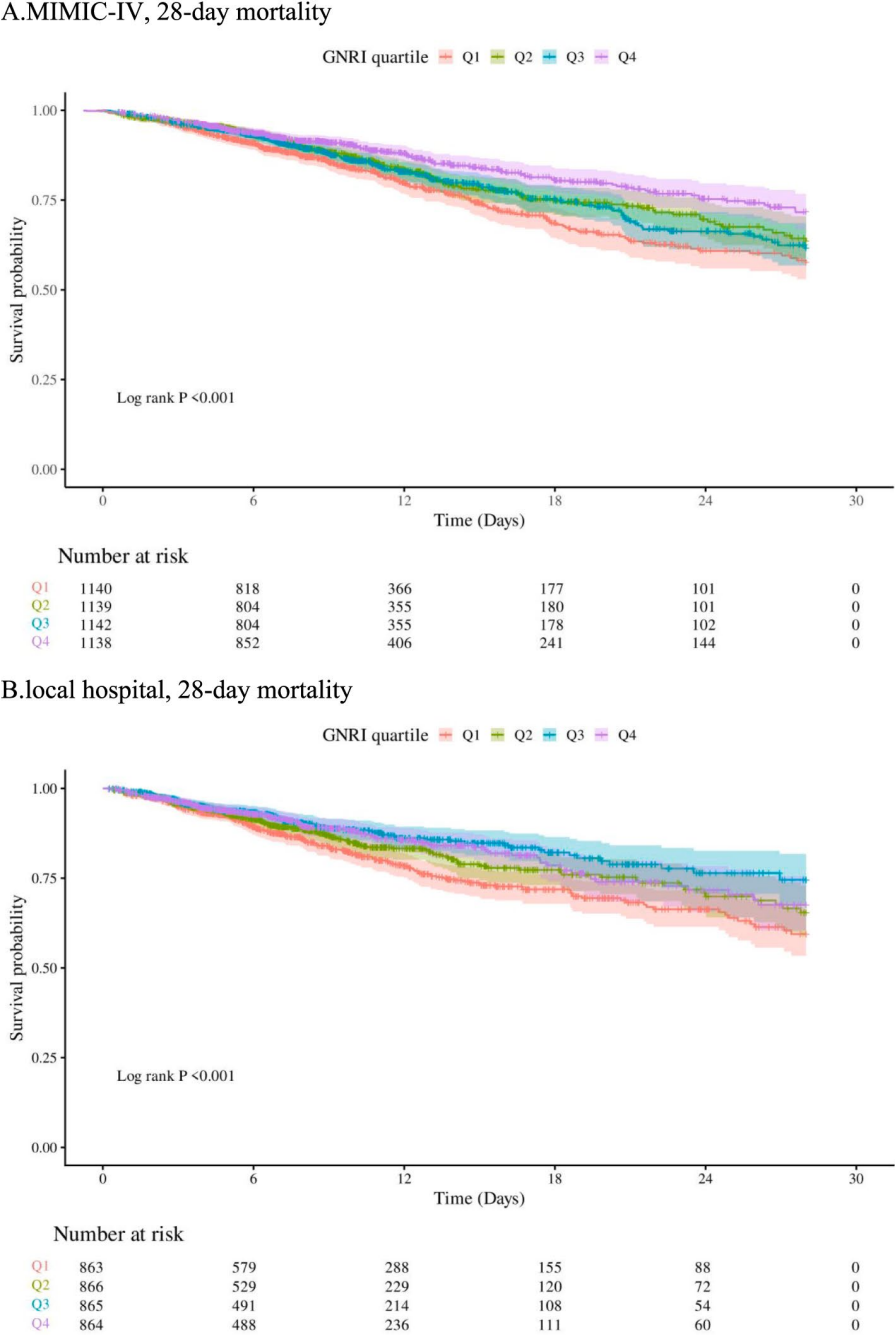
**(A)** Kaplan–Meier curves demonstrating the link between GNRI quartiles and 28-day mortality risk in the MIMIC-IV cohort. **(B)** Kaplan–Meier curves demonstrating the link between GNRI quartiles and 28-day mortality risk in the local hospital cohort. GNRI, Geriatric Nutrition Risk Index.

Prior to constructing the Cox proportional hazards regression models, we completed pre-analytical validation to confirm the appropriateness of the modeling framework. The proportional hazards assumption was assessed by examining three diagnostic tools for 28-day mortality grouped by GNRI quartile: Kaplan–Meier curves, log-minus-log test plots, and residual plots, with crossing of any of these curves considered evidence of assumption violation (Supplementary Figure 2; Figure 1).

To evaluate the linear association between GNRI levels and mortality in patients with AF, we conducted both unadjusted and multivariable Cox proportional hazards regression analyses. Three hierarchical models were constructed for this purpose: Model 1 was the unadjusted model, including only the primary exposure variable with no additional covariates. Model 2 adjusted for an extensive range of demographic, clinical, and treatment-related factors, such as gender, age, race, admission year, initial vital signs at ICU admission (heart rate, mean arterial pressure, respiratory rate, SpO₂, temperature), ICU type (Cardiovascular ICU, Coronary Care Unit, Medical ICU, Surgical ICU, OTHER), SOFA score, Charlson Comorbidity Index, APS III score, life support interventions (CRRT, vasopressors, mechanical ventilation), sepsis diagnosis, prescribed medications (insulin, acetaminophen, diuretics, AF interventions [rate control drugs, cardioversion, rhythm control drugs], dexmedetomidine, statins, phosphorus, magnesium sulfate, potassium, calcium, iron), and comorbid conditions (cardiovascular disease, chronic liver disease, stroke, chronic lung disease, diabetes, cancer, CKD, prior AF). Model 3 built upon Model 2 by adding adjustments for a comprehensive set of laboratory parameters, including white blood cell count, platelet count, hemoglobin, partial pressure of carbon dioxide (pCO₂), partial pressure of oxygen (pO₂), pH, lactate, alanine transaminase (ALT), aspartate transaminase (AST), sodium, creatinine, blood urea nitrogen, potassium, calcium, chloride, glucose, magnesium, and phosphorus.

Kaplan–Meier survival curves were utilized to examine the relationship between elevated GNRI levels and mortality. To further characterize the association between GNRI levels and 28-day mortality risk, we applied Cox proportional hazards regression analysis incorporating restricted cubic splines (RCS). We also performed smooth curve fitting using the penalized spline approach with four knots to better delineate the nature of this relationship.

Analyses were performed using R software (version 4.1.1) and SPSS (version 29.0). A two-tailed *p* < 0.05 was considered statistically significant. All statistical analyses were re-verified after the R code error was identified, and that no other results were affected.

## Results

**Figure 2:**
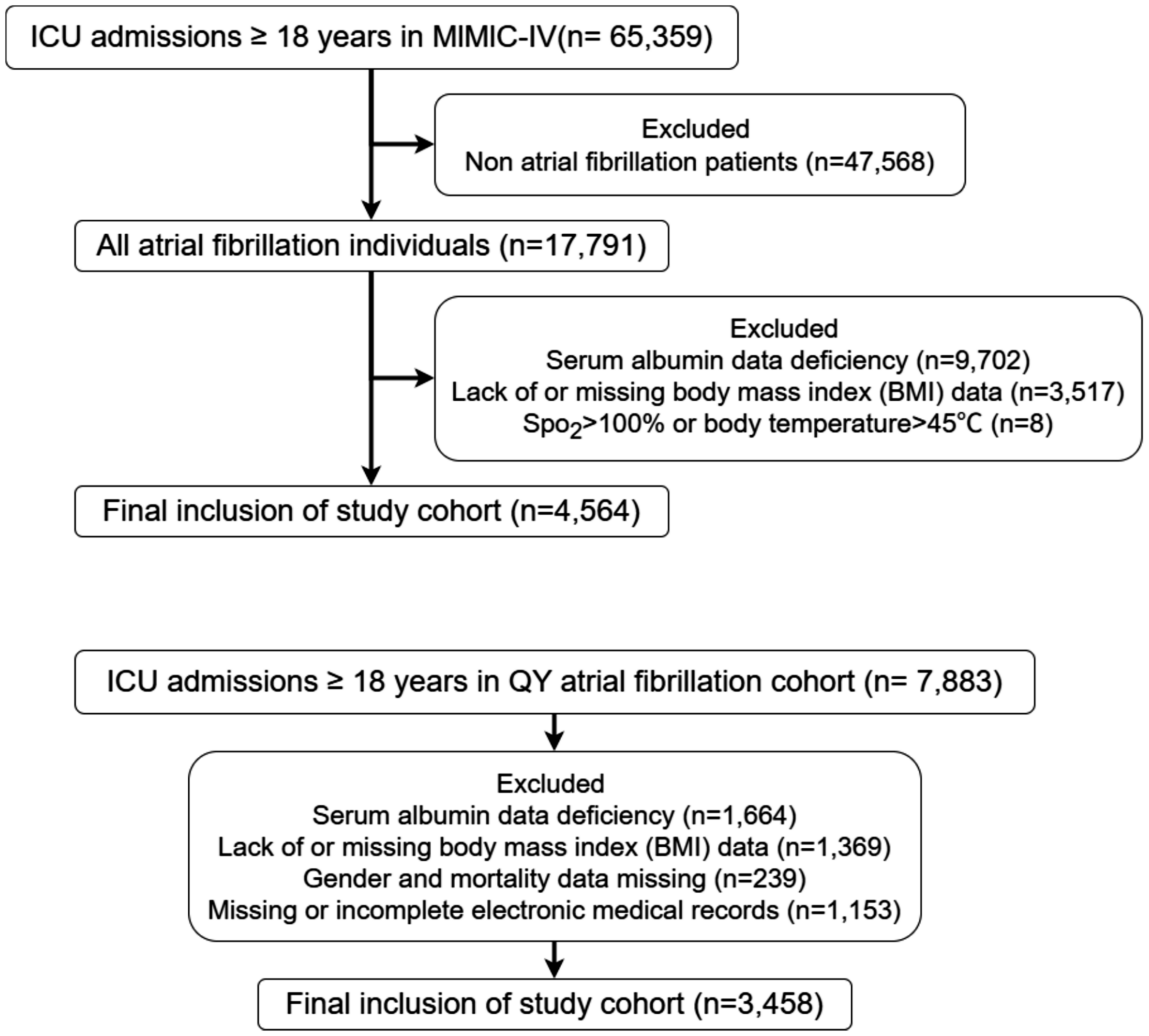
Diagram illustrating the flow of participants through the study population.

The primary cohort of 4,564 AF patients was constructed from the MIMIC-IV database by excluding 60,795 ineligible records from an initial pool of 65,359 entries (Figure 2). Demographic profiles revealed a median (IQR) age of 74.00 (66.00, 82.00) years, 63.8% male patients (*n* = 2,911), and racial/ethnic composition: 4.4% Black (*n* = 201), 2.1% Asian (*n* = 94), 2.0% Hispanic (*n* = 93), 72.3% White (*n* = 3,300), and 19.2% other races/ethnicities (*n* = 876). As GNRI quartiles rose, statistically significant increases were detected in obesity rate, and use of CRRT, mechanical ventilation, vasopressors, diuretics, dexmedetomidine, calcium agents, rate or rhythm control medications, as well as serum levels of potassium, glucose, magnesium, and phosphorus. Conversely, age, the proportion of patients with chronic lung disease, and cancer diagnosis showed significant decreases across higher GNRI quartiles (Table 1).

The local hospital validation analysis enrolled 3,458 patients (Figure 2), with a median age of 66.0 years (IQR, 59.0–73.0) and male representation of 54.3% (*n* = 1,878).

### Mortality risk according to GNRI quartiles

Across the MIMIC-IV cohort, 16.3% of patients experienced 28-day mortality, compared with 14.3% in the local hospital cohort. As GNRI quartiles rose, 28-day mortality rates decreased significantly in the MIMIC-IV cohort (*p* < 0.001): 20.2% in Q1 (GNRI < 90.31), 15.4% in Q2 (90.31 ≤ GNRI < 100.23), 16.4% in Q3 (100.23 ≤ GNRI < 111.28), and 13.3% in Q4 (GNRI ≥ 111.28). The local hospital cohort demonstrated an identical trend (*p* < 0.001), with mortality rates of 20.4% in Q1 (GNRI < 84.92), 14.4% in Q2 (84.92 ≤ GNRI < 96.04), 10.5% in Q3 (96.04 ≤ GNRI < 108.59), and 12.0% in Q4 (GNRI ≥ 108.59) (Table 2).

**Table 2:** Summary of patient outcomes stratified by GNRI quartiles.

	Overall	Q1 GNRI < 90.31	Q2 90.31 ≤ GNRI < 100.23	Q3 100.23 ≤ GNRI < 111.28	Q4 GNRI ≥ 111.28	*p*
Outcomes	*n* = 4,564	*n* = 1,140	*n* = 1,141	*n* = 1,142	*n* = 1,141	
MIMIC-IV						
28 day mortality	745 (16.3)	230 (20.2)	176 (15.4)	187 (16.4)	152 (13.3)	<0.001
28 day ICU mortality	761 (16.7)	237 (20.8)	177 (15.5)	190 (16.6)	157 (13.8)	<0.001
QY cohort	Overall	Q1 GNRI < 84.92	Q2 84.92 ≤ GNRI < 96.04	Q3 96.04 ≤ GNRI < 108.59	Q4 GNRI ≥ 108.59	*p*
	*n* = 3,458	*n* = 863	*n* = 866	*n* = 865	*n* = 864	
28 day mortality	496 (14.3)	176 (20.4)	125 (14.4)	91 (10.5)	104 (12.0)	<0.001
28 day ICU mortality	280 (8.1)	89 (10.3)	69 (8.0)	57 (6.6)	65 (7.5)	0.033

Categorical variables are expressed as count (percentage). Chi-square testing was used to generate P values for comparisons of outcomes between GNRI quartile groups. GNRI, Geriatric Nutrition Risk Index.

In unadjusted Cox regression Model I (Table 3), ascending GNRI quartiles correlated with significantly elevated risks of 28-day mortality and 28-day ICU mortality in MIMIC-IV cohort. In Model II, after controlling for age, gender, race, admission year, type of ICU, initial vital signs at ICU admission, SOFA score, Charlson Comorbidity Index, APS III score, life support interventions, comorbidities, and AF-directed treatments, results for the MIMIC-IV cohort were consistent with Model I. In Model III, after incorporating additional possible confounders, GNRI remained an independent predictor of 28-day mortality (Q4 vs. Q1: HR 0.78, 95% CI 0.62–0.97; *p* = 0.029) and 28-day ICU mortality (Q4 vs. Q1: HR 0.69, 95% CI 0.53–0.91; *p* = 0.009) in the MIMIC-IV cohort. Similarly, this relationship remains robust in the local hospital cohort (Supplementary Tables 1, 2).

**Table 3:** The association between GNRI and mortality risks.

MIMIC-IV	Model 1	Model 2	Model 3
	HR (95%CI) *P*	HR (95%CI) *P*	HR (95%CI) *P*
28 day mortality			
Q1: GNRI < 90.31	1.00 (reference)	1.00 (reference)	1.00 (reference)
Q2: 90.31 ≤ GNRI < 100.23	0.78 (0.64 ~ 0.95) *p* = 0.012	0.93 (0.76 ~ 1.13) *p* = 0.453	0.96 (0.78 ~ 1.18) *p* = 0.704
Q3: 100.23 ≤ GNRI < 111.28	0.82 (0.68 ~ 0.99) *p =* 0.043	1.05 (0.86 ~ 1.28) *p =* 0.651	1.07 (0.87 ~ 1.31) *p =* 0.531
Q4: GNRI ≥ 111.28	0.60 (0.49 ~ 0.74) *P* < 0.001	0.77 (0.62 ~ 0.96) *p =* 0.018	0.78 (0.62 ~ 0.97) *P =* 0.029
28 day ICU mortality			
Q1: GNRI < 90.31	1.00 (reference)	1.00 (reference)	1.00 (reference)
Q2: 90.31 ≤ GNRI < 100.23	0.76 (0.62 ~ 0.92) *p* = 0.005	0.84 (0.68 ~ 1.03) *p* = 0.090	0.87 (0.71 ~ 1.07) *p* = 0.187
Q3: 100.23 ≤ GNRI < 111.28	0.76 (0.63 ~ 0.92) *P* = 0.005	0.85 (0.69 ~ 1.04) *p* = 0.116	0.88 (0.72 ~ 1.09) *p* = 0.239
Q4: GNRI ≥ 111.28	0.55 (0.45 ~ 0.68) *p* < 0.001	0.65 (0.50 ~ 0.85) *p* = 0.002	0.69 (0.53 ~ 0.91) *P* = 0.009

Cox proportional hazards regression was used to construct all study models for MIMIC-IV. Model I: No covariates adjusted. Model II: Adjusted for baseline demographics (gender, age, race, admission year), ICU admission vital signs (heart rate, mean arterial pressure, respiratory rate, SpO₂, temperature), ICU setting (Cardiovascular ICU, Coronary Care Unit, Medical ICU, Surgical ICU, OTHER), illness severity scores (SOFA, Charlson Comorbidity Index, APS III), invasive interventions (CRRT, vasopressors, mechanical ventilation), sepsis status, pharmacotherapies [insulin, acetaminophen, diuretics, AF management strategies (rate control drugs, cardioversion, rhythm control agents), dexmedetomidine, statins, mineral supplements (phosphorus, magnesium sulfate, potassium, calcium), iron], and pre-existing comorbidities (cardiovascular disease, chronic liver disease, stroke, chronic lung disease, diabetes, cancer, CKD, prior AF). Model III: Adjusted for hematologic parameters (white blood cell count, platelet count, hemoglobin), blood gas markers (pCO₂, pO₂, pH, lactate), hepatic enzymes (ALT, AST), and serum biochemistries (sodium, creatinine, blood urea nitrogen, potassium, calcium, chloride, glucose, magnesium, phosphorus). GNRI, Geriatric Nutrition Risk Index; HR, hazard ratio; CI, confidence interval.

Kaplan–Meier survival analysis identified progressive variability in survival rates across GNRI quartiles (Figure 1; Supplementary Figure 1). The higher GNRI group demonstrated a higher survival rate relative to the lower GNRI group (MIMIC-IV: log-rank *p* < 0.001; local hospital: log-rank *p* < 0.001).

**Figure 3:**
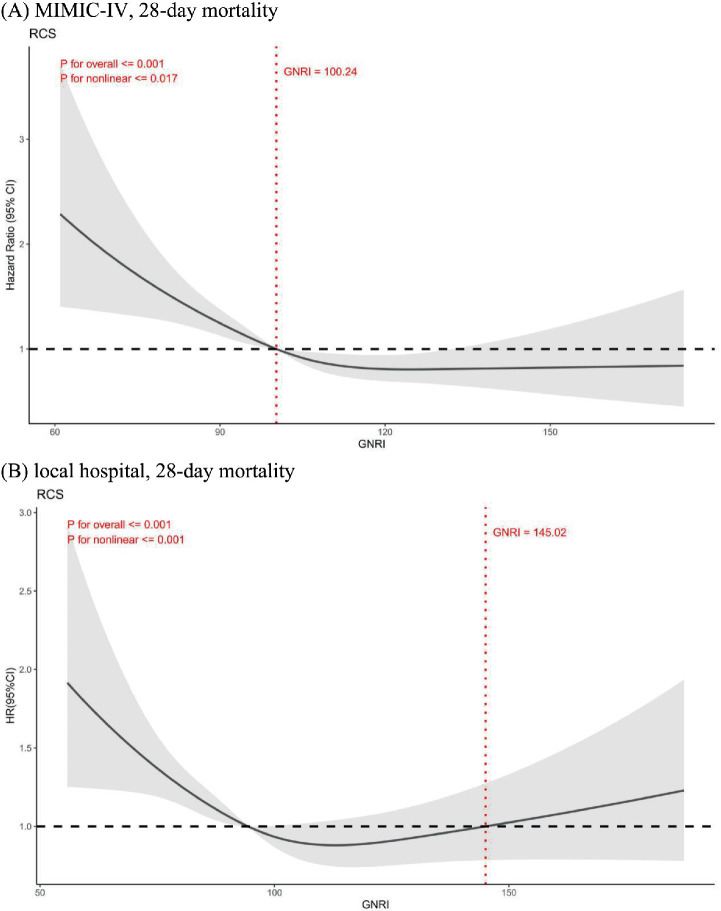
RCS models adjusted depict the link between GNRI and 28-day mortality risk **(A)** in the MIMIC-IV cohort and **(B)** in the local hospital cohort patients. RCS, restricted cubic spline; GNRI, Geriatric Nutrition Risk Index.

Restricted cubic spline were employed to evaluate the association between GNRI levels and mortality risks for 2 cohorts. As illustrated in Figure 3, a statistically significant relationship was observed between GNRI levels and 28-day mortality (p for overall association < 0.001, p for nonlinearity = 0.017) among individuals with AF for MIMIC-IV. The analysis identified a first inflection point at a GNRI level of 100.24; beyond this point, higher GNRI levels were associated with a progressively lower probability of mortality (Figure 3). Similar trends (28-day mortality: p for overall association <0.001, p for nonlinearity <0.001) and inflection points (145.02) can also be observed in the local hospital cohort.

### Subgroup analysis

**Figure 4:**
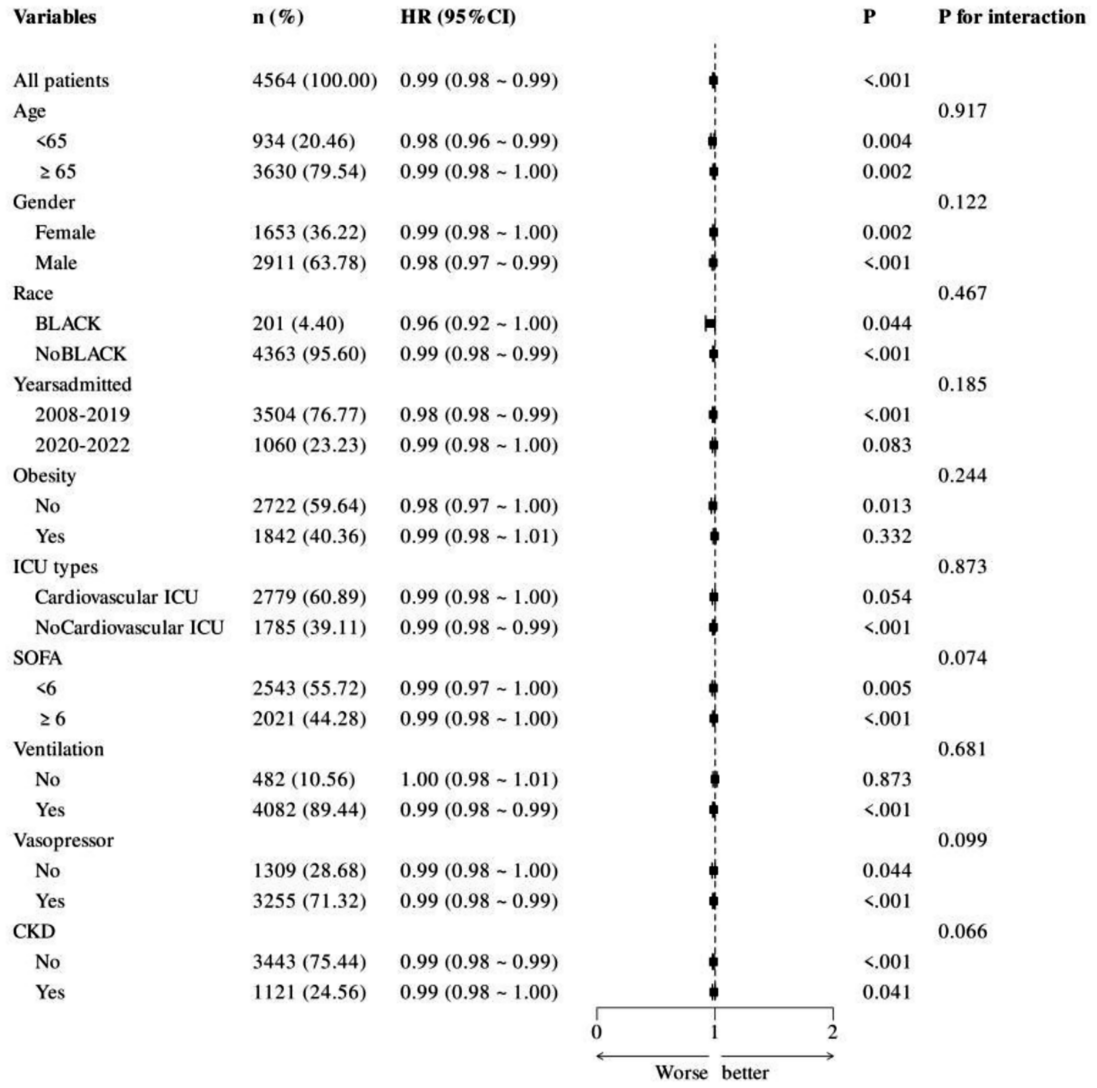
Stratified subgroup analysis examining the correlation between increased GNRI levels and 28-day mortality risk. Adjustments in Cox proportional hazards regression models included initial ICU vital signs (heart rate, mean arterial pressure, respiratory rate, SpO₂, temperature), illness severity metrics (Charlson Comorbidity Index, APS III), invasive interventions (vasopressors, mechanical ventilation), medication regimens [insulin, acetaminophen, diuretics, atrial fibrillation management approaches (rate control drugs, cardioversion, rhythm control agents), dexmedetomidine, statins, mineral supplements (phosphorus, magnesium sulfate, potassium, calcium), iron], chronic comorbidities (cardiovascular disease, chronic liver disease, stroke, chronic lung disease, diabetes, cancer), hematologic indices (white blood cell count, platelet count, hemoglobin), blood gas parameters (pCO₂, pO₂, pH, lactate), liver function tests (ALT, AST), and serum chemistry panels (sodium, creatinine, blood urea nitrogen, potassium, calcium, chloride, glucose, magnesium, phosphorus). GNRI, Geriatric Nutrition Risk Index; SOFA, sequential organ failure assessment.

Subgroup analyses and interaction effect evaluations were carried out to characterize the relationship between GNRI levels and 28-day mortality risk across multiple patient subgroups, stratified by age, gender, race, admission year, ICU setting, SOFA score, mechanical ventilation status, vasopressor therapy, cardiac surgery, CRRT initiation, sepsis diagnosis, new-onset vs. prior AF, and CKD (Figure 4; Table 4).

**Table 4:** Total GNRI levels and 28 day mortality in critically ill patients with prior or New AF.

MIMIC-IV	Model 1	Model 2	Model 3
	HR (95%CI) *P*	HR (95%CI) *P*	HR (95%CI) *P*
28 day mortality			
All	0.99 (0.99 ~ 0.99) *p* < 0.001	0.99 (0.99 ~ 0.99) *p* < 0.001	0.99 (0.98 ~ 0.99) *p* < 0.001
New-onset AF	0.99 (0.98 ~ 0.99) *p* < 0.001	0.99 (0.98 ~ 0.99) *p* < 0.001	0.99 (0.99 ~ 0.99) *P =* 0.009
Prior AF	1.00 (0.99 ~ 1.00) *p =* 0.178	0.99 (0.99 ~ 1.00) *p =* 0.072	1.00 (0.99 ~ 1.01) *p =* 0.761
Sepsis			
All	0.99 (0.99 ~ 0.99) *P* = 0.005	0.99 (0.99 ~ 0.99) *p* < 0.001	0.99 (0.99 ~ 0.99) *p* < 0.001
New-onset AF			
Continuous	0.99 (0.98 ~ 0.99) *p* < 0.001	0.99 (0.98 ~ 0.99) *p* = 0.001	0.99 (0.98 ~ 0.99) *P =* 0.028
Q1: GNRI < 90.85	1.00 (reference)	1.00 (reference)	1.00 (reference)
Q2: 90.85 ≤ GNRI < 100.77	0.75 (0.56 ~ 1.00) *p* = 0.052	0.90 (0.67 ~ 1.22) *p* = 0.509	0.91 (0.67 ~ 1.24) *p* = 0.541
Q3: 100.77 ≤ GNRI < 111.61	0.87 (0.65 ~ 1.15) *p =* 0.316	0.94 (0.70 ~ 1.27) *p =* 0.684	0.94 (0.70 ~ 1.28) *p =* 0.711
Q4: GNRI ≥ 111.61	0.56 (0.41 ~ 0.76) *p* < 0.001	0.64 (0.46 ~ 0.91) *p =* 0.012	0.66 (0.46 ~ 0.93) *p =* 0.019
Prior AF	1.00 (0.99 ~ 1.00) *p =* 0.540	0.99 (0.99 ~ 1.00) *p =* 0.118	1.00 (0.99 ~ 1.00) *p =* 0.313
CRRT			
All	1.00 (0.99 ~ 1.01) *p =* 0.859	1.00 (0.99 ~ 1.01) *p =* 0.706	1.00 (0.99 ~ 1.01) *p =* 0.846
New-onset AF	1.01 (0.99 ~ 1.02) *p =* 0.859	1.01 (0.99 ~ 1.02) *p =* 0.262	1.01 (1.00 ~ 1.02) *p =* 0.198
Prior AF	0.99 (0.97 ~ 1.00) *p =* 0.133	0.99 (0.97 ~ 1.00) *p =* 0.092	0.99 (0.97 ~ 1.00) *p =* 0.109
Cardiac surgery			
All	1.00 (0.98 ~ 1.02) *p =* 0.817	1.00 (0.97 ~ 1.02) *p =* 0.878	1.01 (0.99 ~ 1.04) *p =* 0.320
New-onset AF	0.99 (0.97 ~ 1.01) *p =* 0.308	0.99 (0.97 ~ 1.02) *p =* 0.579	0.99 (0.97 ~ 1.01) *p =* 0.479
Prior AF	1.07 (1.01 ~ 1.13) *p =* 0.018	1.06 (1.01 ~ 1.11) *p =* 0.019	1.05 (0.97 ~ 1.13) *p =* 0.201

GNRI, Geriatric Nutrition Risk Index; HR, hazard ratio; CI, confidence interval; CRRT, continuous renal replacement therapy; AF, atrial fibrillation.

Notably, this association was absent in obese, prior AF, cardiac surgery, and CRRT patients. In patients with new-onset AF complicated by sepsis, however, the association between GNRI levels and 28-day mortality remained statistically robust in the MIMIC-IV cohort: continuous GNRI (Model III): HR 0.99, 95% CI 0.98–0.99; *p* = 0.028; quartile GNRI (Model III, Q4 vs. Q1): HR 0.66, 95% CI 0.46–0.93; *p* = 0.019; In addition, no such association was found in the local hospital cohort (continuous GNRI, HR 1.00, 95% CI 0.99–1.00; *p* = 0.391) (Supplementary Table 3). In contrast, no significant relationship was detected between GNRI levels and mortality in sepsis patients with prior AF (Figure 4; Table 4).

## Discussion

This cohort study indicated that higher GNRI levels were associated with reduced 28-day mortality and 28-day ICU mortality, with consistent results replicated across two hospital cohorts (MIMIC-IV and local hospital cohorts). Notably, this beneficial association was not detected in obese patients, those with prior AF, individuals undergoing cardiac surgery or admitted to the cardiovascular ICU, or CRRT-treated patients. Conversely, in patients with new-onset AF complicated by sepsis, the association between GNRI levels and 28-day mortality was inconsistent in both cohorts. No significant correlation was observed between GNRI levels and mortality in sepsis patients with prior AF. Kaplan–Meier survival curves demonstrated that patients with elevated GNRI had lower 28-day mortality rates in both cohorts. RCS analysis confirmed a significant relationship between GNRI levels and mortality risk.

Findings from our investigative analysis offer distinct perspectives on nutritional status and clinical outcomes in critically ill patients. We are the first to establish that higher GNRI values correlate significantly with improved prognostic trajectories in critically ill patients presenting with new-onset AF. Numerous prior studies have examined the relationship between GNRI and prognosis in critically ill populations. Mei et al. reported that lower GNRI scores were associated with adverse outcomes in 2,824 elderly ICU patients with COPD (32). Xiong and colleagues found that reduced GNRI was strongly linked to decreased survival in 4,575 ICU patients with AKI. Tang et al. demonstrated an inverse correlation between GNRI and in-hospital as well as 30-day mortality, even after accounting for confounders, in 5,506 critically ill patients with acute myocardial infarction. Gao et al. identified that higher GNRI was negatively associated with increased short-term mortality in 1,244 critically ill patients with cerebral injury. Zhang and associates observed that higher GNRI correlated with elevated all-cause mortality in critically ill older patients with CAP (33). Similarly, suboptimal nutritional status is closely associated with poor prognosis in sepsis. In contrast to these prior analyses, our study focused exclusively on critically ill patients with AF. In non-ICU settings, research has also documented a strong link between poor nutritional status and AF onset or progression. One study identified GNRI as a predictor of arrhythmia recurrence following catheter ablation for AF (34). Another investigation highlighted GNRI’s potential role as a novel risk factor for non-valvular AF development in heart failure patients (35). Additionally, a study explored the relationship between GNRI and long-term outcomes in hospitalized AF patients (36). Malnutrition, as determined by the Mini-Nutritional Assessment-Short Form (MNA-SF), represents an independent risk factor for all-cause mortality in elderly patients with nonvalvular AF (37).

Although our investigation failed to define a causal mechanism connecting inadequate nutritional status to poor prognosis in newly diagnosed AF patients, we propose some potential biological links. Long-standing AF can lead to cardiac decompensation, ischemic stroke, and systemic embolism, all of which diminish physical function and reduce mobility. These complications disrupt nutritional intake and utilization, gradually progressing to frailty and malnutrition. Notably, studies in underweight heart failure patients have shown that increasing BMI correlates with reduced mortality risk, implying that optimized nutritional interventions may lower all-cause mortality in elderly AF patients—though this requires confirmation in dedicated clinical trials (38). Chronic low-grade inflammation may act as a key intermediary between AF and malnutrition. Chronic illnesses induce persistent systemic inflammation, characterized by elevated levels of proinflammatory cytokines including interleukin-6 (IL-6), tumor necrosis factor-alpha (TNF-*α*), and C-reactive protein (CRP). These mediators exert widespread metabolic effects, increasing protein breakdown, inhibiting fat synthesis, and driving metabolic dysregulation that culminates in malnutrition (39). These proinflammatory mediators also exert profound effects on the pathogenesis and progression of AF (40, 41).

Notably, this beneficial association is absent in critically ill patients with pre-existing AF. Differentiating the pathological characteristics of critically ill patients with pre-existing AF versus new-onset AF is essential for targeted clinical management. Pre-existing AF is inherently a chronic arrhythmia, observed in patients admitted across a broad spectrum of critical care settings—including medical intensive care units (MICUs), cardiac and non-cardiac surgical ICUs, cardiovascular intensive care units (CVICUs), coronary care units (CCUs), and neurocritical care units (NCICUs). The pathophysiology of AF involves a complex interplay of factors: advanced age, diabetes mellitus, chronic heart failure, chronic kidney disease, activation of TGF-β1, renin-angiotensin system dysregulation, inflammation and reactive oxygen species (ROS) generation, tachycardia-induced cardiac remodeling, and progressive electrical or structural myocardial changes. Critically, the mechanisms driving AF development and persistence may not overlap with those by which malnutrition contributes to this AF risk.

Our findings demonstrate no significant association between malnutrition and outcomes in patients undergoing cardiac surgery or admitted to cardiovascular ICUs. This observation suggests that the inflammatory potential of malnutrition may be insufficiently impactful in this cohort, given that surgical alterations to cardiac structure play a predominant role in driving the pathophysiology of AF (42). Notably, our analysis found no significant link between nutritional status and outcomes in AF patients receiving CRRT. This lack of association is likely due to the fact that poor nutritional status may have limited impact on the pathophysiology of AF in this population, as concomitant fluid and electrolyte disorders—common in patients requiring CRRT or with AKI—appear to drive the underlying mechanisms instead (43, 44). Our data demonstrate no association between GNRI levels and negative prognostic outcomes in obese patients, suggesting two plausible mechanisms: First, enhanced nutritional status could effectively offset the detrimental effects of malnutrition in ICU patients with AF; second, the underrepresentation of malnutrition in obese cohorts may underlie the lack of statistical significance detected in our results.

## Limitations

Several important limitations of this study should be acknowledged. First, although we controlled for multiple confounding variables, the retrospective nature of the research inherently leaves some residual confounding unaddressed. Second, nutritional status was only measured at baseline, making it impossible to explore how longitudinal changes in nutritional status correlate with study outcomes. Third, we did not adjust for inflammatory markers or other biological mediators that may underpin the nutrition-outcome association. Fourth, anthropometric assessments such as body composition (e.g., body fat percentage versus lean body mass) and physical activity data were not recorded—information that could have clarified the mechanisms underlying the observed nutrition-outcome relationship. Lastly, patient selection criteria for AF trials may differ according to nutritional status, thereby contributing to heterogeneous outcome risks among study participants.

## Conclusion

Results from this two-cohort analysis demonstrate that higher GNRI scores are associated with reduced mortality risk in critically ill patients with atrial fibrillation across two independent ICU cohorts. This favorable association was not observed in patients with preexisting AF, supporting the external generalizability of the core findings. In the subgroup of patients with sepsis and new-onset atrial fibrillation, the association between GNRI and mortality was not replicated in the external validation cohort. These results suggest that well-preserved nutritional status may confer a protective survival benefit in critically ill patients with new-onset AF. Prospective randomized controlled trials are required to validate these observations before clinical application can be considered.

## Data Availability

The original contributions presented in the study are included in the article/Supplementary material, further inquiries can be directed to the corresponding author.
