# Supplementary material for: The geriatric nutritional risk index as a prognostic risk factor for critically ill patients with atrial fibrillation: a retrospective study based on MIMIC-IV and local hospital cohort external validation
Source: Front Nutr. 2026 Jul 6;13:1837424. doi: 10.3389/fnut.2026.1837424 (PMC13381237; doi:10.3389/fnut.2026.1837424)
Supplement: Supplementary file 1 [file Table_1.docx]

**Supplementary Table 1.**

Patient baseline characteristics in the local hospital cohort stratified by GNRI quartiles

**Supplementary Table 2.**

The association between GNRI and mortality risks in the local hospital cohort

**Supplementary Table 3.**

Total GNRI levels and 28-day mortality in sepsis patients with new-onset AF in the local hospital cohort

**Supplementary Figure 1.**

Kaplan-Meier curves demonstrating the link between GNRI quartiles and 28-day mortality risk in the MIMIC-IV cohort excluding patients who died within the first day of hospitalization

**Supplementary Figure 2.**

Proportional hazards assumption analysis

**Supplementary Table 1.**

Patient baseline characteristics in the local hospital cohort stratified by GNRI quartiles

|  | (Median [IQR]) |  |  |  |  |  |
| --- | --- | --- | --- | --- | --- | --- |
|  | **Overall** | **Q1** | **Q2** | **Q3** | **Q4** | *p* |
| **Variable (%)** | **n=3458** | **n=863** | **n=866** | **n=863** | **n=866** |  |
| Age, ys | 66.00 [59.00, 73.00] | 70.00 [63.00, 78.00] | 65.00 [59.00, 73.00] | 65.00 [58.00, 73.00] | 63.00 [57.00, 70.00] | <0.001 |
| Gender |  |  |  |  |  |  |
| Female | 1580 (45.7) | 429 (49.7) | 393 (45.4) | 374 (43.3) | 384 (44.3) | 0.042 |
| Male | 1878 (54.3) | 434 (50.3) | 473 (54.6) | 489 (56.7) | 482 (55.7) |  |
| BMI,kg/m^2^ | 27.60 [23.60, 33.30] | 22.20 [19.80, 24.65] | 25.30 [23.22, 28.10] | 29.20 [26.60, 32.40] | 37.60 [33.90, 43.60] | <0.001 |
| Heart rate, beats/min | 90.00 [75.00, 109.00] | 94.00 [80.00, 113.00] | 90.00 [76.00, 110.00] | 88.00 [74.00, 107.50] | 88.50 [74.00, 105.00] | <0.001 |
| Mean artery pressure,mm Hg | 81.00 [60.00, 101.00] | 82.00 [60.00, 101.00] | 81.00 [60.25, 101.00] | 80.00 [62.00, 102.00] | 80.00 [59.00, 100.00] | 0.553 |
| Respiratory rate, breaths/min | 20.00 [17.00, 24.00] | 21.00 [18.00, 26.00] | 21.00 [17.00, 25.00] | 20.00 [16.00, 24.00] | 20.00 [17.00, 24.00] | <0.001 |
| SpO2 level, % | 97.00 [95.00, 99.00] | 97.00 [95.00, 100.00] | 98.00 [95.00, 100.00] | 97.00 [95.00, 99.00] | 97.00 [95.00, 99.00] | <0.001 |
| Temperature, °C | 36.70 [36.40, 37.00] | 36.70 [36.30, 37.00] | 36.70 [36.40, 37.00] | 36.70 [36.40, 37.00] | 36.70 [36.40, 37.00] | 0.33 |
| APS III score | 87.00 [46.00, 128.00] | 90.00 [46.00, 128.00] | 84.50 [43.00, 128.75] | 86.00 [48.50, 127.50] | 88.00 [46.00, 127.00] | 0.84 |
| SOFA score | 4.00 [2.00, 7.00] | 4.00 [2.00, 7.00] | 4.00 [2.00, 6.75] | 4.00 [2.00, 6.00] | 4.00 [2.00, 7.00] | 0.087 |
| Ventilation |  |  |  |  |  | 0.245 |
| No | 2473 (71.5) | 602 (69.8) | 609 (70.3) | 624 (72.3) | 638 (73.7) |  |
| Yes | 985 (28.5) | 261 (30.2) | 257 (29.7) | 239 (27.7) | 228 (26.3) |  |
| vasopressor |  |  |  |  |  | 0.002 |
| No | 2538 (73.4) | 592 (68.6) | 645 (74.5) | 657 (76.1) | 644 (74.4) |  |
| Yes | 920 (26.6) | 271 (31.4) | 221 (25.5) | 206 (23.9) | 222 (25.6) |  |
| CRRT |  |  |  |  |  | 0.331 |
| No | 3281 (94.9) | 826 (95.7) | 814 (94.0) | 815 (94.4) | 826 (95.4) |  |
| Yes | 177 ( 5.1) | 37 ( 4.3) | 52 ( 6.0) | 48 ( 5.6) | 40 ( 4.6) |  |
| Cancer |  |  |  |  |  | <0.001 |
| No | 3198 (92.5) | 764 (88.5) | 805 (93.0) | 807 (93.5) | 822 (94.9) |  |
| Yes | 260 ( 7.5) | 99 (11.5) | 61 ( 7.0) | 56 ( 6.5) | 44 ( 5.1) |  |
| CKD |  |  |  |  |  | 0.313 |
| No | 2783 (80.5) | 711 (82.4) | 688 (79.4) | 698 (80.9) | 686 (79.2) |  |
| Yes | 675 (19.5) | 152 (17.6) | 178 (20.6) | 165 (19.1) | 180 (20.8) |  |
| COPD |  |  |  |  |  |  |
| No | 2717 (78.6) | 661 (76.6) | 675 (77.9) | 713 (82.6) | 668 (77.1) | 0.009 |
| Yes | 741 (21.4) | 202 (23.4) | 191 (22.1) | 150 (17.4) | 198 (22.9) |  |
| Diabetes |  |  |  |  |  | <0.001 |
| No | 2988 (86.4) | 794 (92.0) | 771 (89.0) | 745 (86.3) | 678 (78.3) |  |
| Yes | 470 (13.6) | 69 ( 8.0) | 95 (11.0) | 118 (13.7) | 188 (21.7) |  |
| Cardiovascular disease |  |  |  |  |  | 0.603 |
| No | 1530 (44.2) | 387 (44.8) | 388 (44.8) | 389 (45.1) | 366 (42.3) |  |
| Yes | 1928 (55.8) | 476 (55.2) | 478 (55.2) | 474 (54.9) | 500 (57.7) |  |
| Chronic lung disease |  |  |  |  |  | 0.001 |
| No | 2615 (75.6) | 624 (72.3) | 644 (74.4) | 694 (80.4) | 653 (75.4) |  |
| Yes | 843 (24.4) | 239 (27.7) | 222 (25.6) | 169 (19.6) | 213 (24.6) |  |
| Chronic neurologic disease |  |  |  |  |  | 0.18 |
| No | 3042 (88.0) | 752 (87.1) | 758 (87.5) | 752 (87.1) | 780 (90.1) |  |
| Yes | 416 (12.0) | 111 (12.9) | 108 (12.5) | 111 (12.9) | 86 ( 9.9) |  |
| Sepsis |  |  |  |  |  | <0.001 |
| No | 2492 (72.1) | 541 (62.7) | 636 (73.4) | 682 (79.0) | 633 (73.1) |  |
| Yes | 966 (27.9) | 322 (37.3) | 230 (26.6) | 181 (21.0) | 233 (26.9) |  |
| **Laboratory tests** |  |  |  |  |  |  |
| Potassium level, mEq/L | 4.15 [3.80, 4.55] | 4.10 [3.75, 4.50] | 4.13 [3.80, 4.55] | 4.15 [3.80, 4.53] | 4.20 [3.85, 4.60] | 0.007 |
| Lactate level, mg/dL | 2.49 [2.00, 2.49] | 2.49 [1.70, 2.49] | 2.49 [2.00, 2.49] | 2.49 [2.49, 2.49] | 2.49 [2.30, 2.49] | 0.018 |
| Ureanitrogen level, mg/dL | 26.00 [17.00, 42.00] | 26.00 [18.00, 42.00] | 28.00 [17.25, 43.75] | 25.00 [17.00, 40.00] | 26.00 [17.00, 43.00] | 0.287 |
| Creatinine level, mg/dL | 1.20 [0.83, 1.90] | 1.10 [0.73, 1.73] | 1.23 [0.82, 1.95] | 1.20 [0.88, 1.90] | 1.27 [0.90, 2.04] | <0.001 |
| Albumin, g/dL | 3.00 [2.00, 4.00] | 3.00 [2.00, 4.00] | 3.00 [2.00, 4.07] | 3.10 [2.10, 4.10] | 3.00 [1.90, 4.00] | 0.307 |

**Supplementary Table 2.**

The association between GNRI and mortality risks in the local hospital cohort.

| **QY cohort** | **Model 1** | **Model 2** | **Model 3** |
| --- | --- | --- | --- |
|  | **HR (95%CI) *P*** | **HR (95%CI) *P*** | **HR (95%CI) *P*** |
| **28 day mortality** |  |  |  |
| Q1: GNRI < 84.92 | 1.00 (Reference) | 1.00 (Reference) | 1.00 (Reference) |
| Q2: 84.92 ≤ GNRI < 96.04 | 0.80 (0.63 ~ 1.00)  *P*=0.052 | 0.81 (0.64 ~ 1.03)  *P*=0.230 | 0.77 (0.61 ~ 0.98)  *P*=0.03 |
| Q3: 96.04 ≤ GNRI < 108.59 | 0.61 (0.48 ~ 0.79)  *P*<.001 | 0.62 (0.48 ~ 0.80)  *P*<.001 | 0.64 (0.49 ~ 0.84)  *P=*0.001 |
| Q4: GNRI ≥ 108.59 | 0.70 (0.55 ~ 0.89)  *P=*0.003 | 0.70 (0.54 ~ 0.90)  *P=*0.005 | 0.70 (0.54 ~ 0.91)  *P=*0.007 |
| Continuous variable | 0.99 (0.99 ~ 0.99)  *P*<.001 | 0.99 (0.99 ~ 0.99)  *P*=0.004 | 0.99 (0.99 ~ 0.99)  *P*=0.026 |
| GNRI < 90.00 | 1.00 (Reference) | 1.00 (Reference) | 1.00 (Reference) |
| GNRI ≥ 90.00 | 0.73 (0.61 ~ 0.87)  *P*<.001 | 0.81 (0.68 ~ 0.97)  *P*=0.022 | 0.80 (0.65 ~ 0.99)  *P*=0.038 |
| **28 day ICU mortality** |  |  |  |
| Q1: GNRI < 84.92 | 1.00 (Reference) | 1.00 (Reference) | 1.00 (Reference) |
| Q2: 84.92 ≤ GNRI < 96.04 | 0.79 (0.58 ~ 1.08)  *P*=0.141 | 0.79 (0.58 ~ 1.08)  *P*=0.141 | 0.74 (0.54 ~ 1.02)  *P*=0.07 |
| Q3: 96.04 ≤ GNRI < 108.59 | 0.67 (0.45 ~ 0.90)  *P*=0.011 | 0.67 (0.48 ~ 0.93)  *P*=0.016 | 0.68 (0.47 ~ 0.96)  *P*=0.02 |
| Q4: GNRI ≥ 108.59 | 0.71 (0.52 ~ 0.98)  *P*=0.036 | 0.71 (0.52 ~ 0.98)  *P*=0.036 | 0.74 (0.52 ~ 1.04)  *P*=0.08 |
| Continuous variable | 0.99 (0.98 ~ 0.99)  *P*=0.004 | 0.99 (0.98 ~ 0.99)  *P*=0.008 | 0.99 (0.99 ~ 0.99)  *P*=0.045 |
| GNRI < 90.00 | 1.00 (Reference) | 1.00 (Reference) | 1.00 (Reference) |
| GNRI ≥ 90.00 | 0.74 (0.59 ~ 0.93)  *P*=0.009 | 0.74 (0.59 ~ 0.93)  *P*=0.010 | 0.77 (0.60 ~ 0.98)  *P*=0.035 |

**Supplementary Table 3.**

Total GNRI levels and 28-day mortality in sepsis patients with new-onset AF in the local hospital cohort

| **QY cohort** | **HR (95%CI)** | ***P*** |
| --- | --- | --- |
| **28 day mortality** |  |  |
| Continuous variable |  |  |
| Sepsis new-onset AF | 1.00 (0.99 ~ 1.00) | 0.391 |

**Supplementary Figure 1.**

Kaplan-Meier curves demonstrating the link between GNRI quartiles and 28-day mortality risk in the MIMIC-IV cohort excluding patients who died within the first day of hospitalization

| MIMIC-IV, 28-day mortality |
| --- |
| 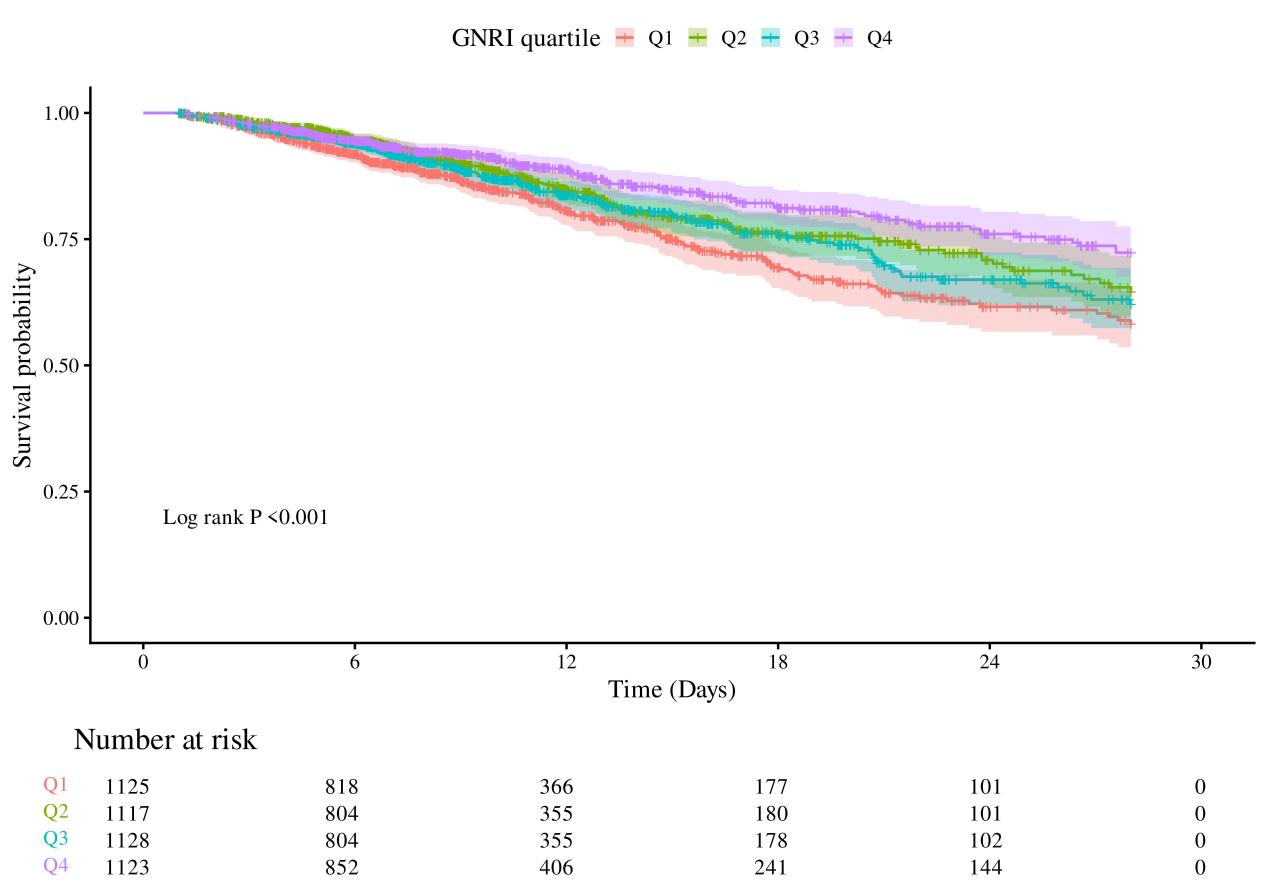 |

**Supplementary Figure 2.**

Proportional hazards assumption analysis

| MIMIC-IV |  |
| --- | --- |
| A. Log-minus-log test plots | B. Residual plots |
| 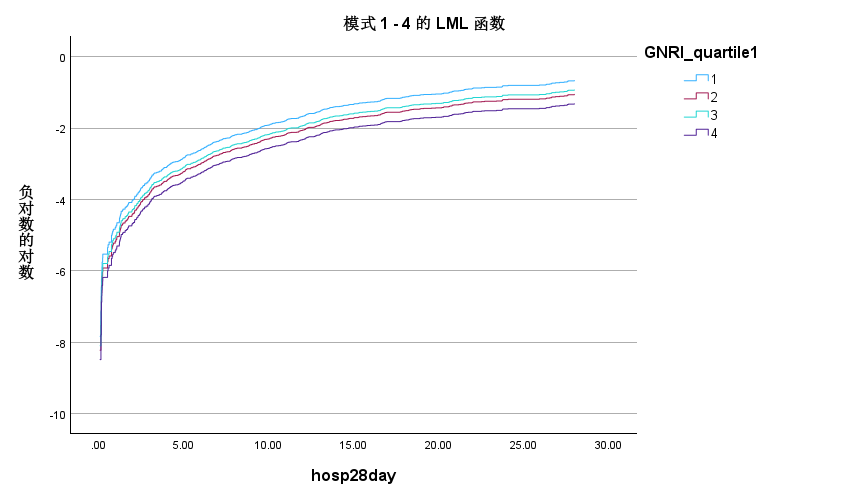 | 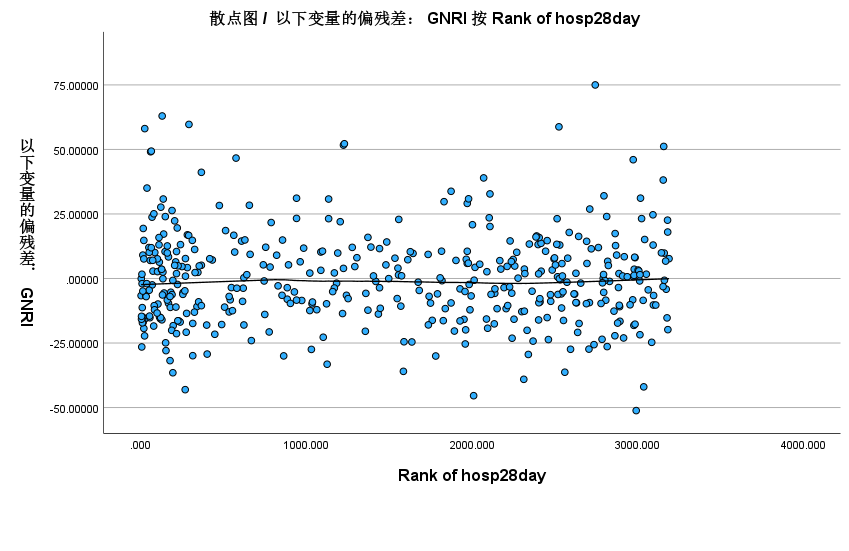 |
| Local hospital cohort |  |
| C. Log-minus-log test plots | D. Residual plots |
| 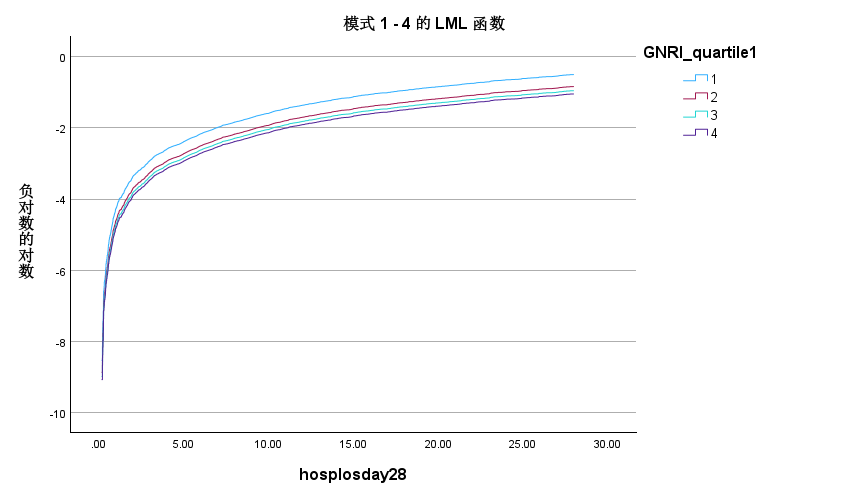 | 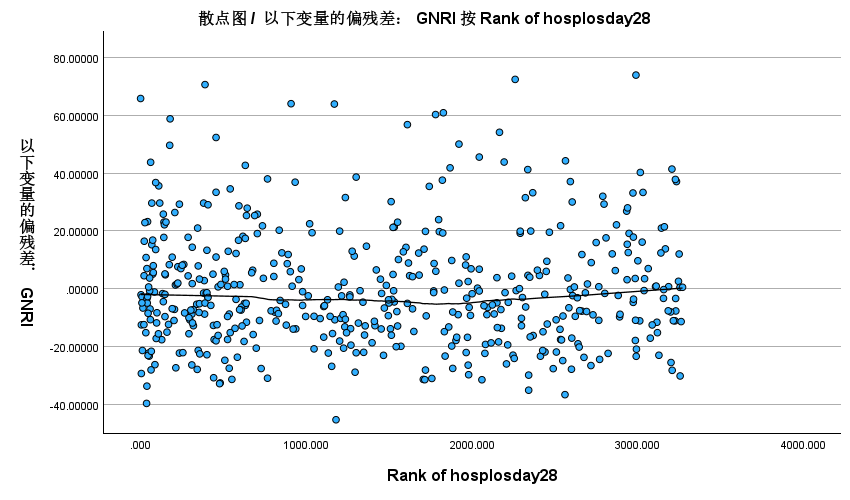 |
